# Functional and comparative genome analysis of novel virulent actinophages belonging to *Streptomyces flavovirens*

**DOI:** 10.1186/s12866-017-0940-7

**Published:** 2017-03-03

**Authors:** A. Sharaf, F. Mercati, I. Elmaghraby, R. M. Elbaz, E. M. Marei

**Affiliations:** 1 Genetic Department, Faculty of Agriculture, Ain Shams University, Cairo, 11241 Egypt; 2 Institute of Parasitology, Biology Centre, Czech Academy of Sciences, 37005 České Budějovice, Czechia; 3 Institute of Biosciences and Bioresources (IBBR), National Research Council (CNR) of Italy, 90129 Palermo, Italy; 4 Central Lab. of Organic Agriculture, Agricultural Research Center, Giza, 12619 Egypt; 5 Botany and Microbiology Department, Faculty of Science, Helwan University, Ain-Helwan, Cairo, 11970 Egypt; 6 Microbiology Department, Faculty of Agriculture, Ain Shams University, Cairo, 11241 Egypt

**Keywords:** Bacteriophage, Biological stability, Whole genome sequence, NGS, Comparative genomics

## Abstract

**Background:**

Next Generation Sequencing (NGS) technologies provide exciting possibilities for whole genome sequencing of a plethora of organisms including bacterial strains and phages, with many possible applications in research and diagnostics. No *Streptomyces flavovirens* phages have been sequenced to date; there is therefore a lack in available information about *S. flavovirens* phage genomics. We report biological and physiochemical features and use NGS to provide the complete annotated genomes for two new strains (Sf1 and Sf3) of the virulent phage *Streptomyces flavovirens*, isolated from Egyptian soil samples.

**Results:**

The *S. flavovirens* phages (Sf1 and Sf3) examined in this study show higher adsorption rates (82 and 85%, respectively) than other actinophages, indicating a strong specificity to their host, and latent periods (15 and 30 min.), followed by rise periods of 45 and 30 min. As expected for actinophages, their burst sizes were 1.95 and 2.49 virions per mL. Both phages were stable and, as reported in previous experiments, showed a significant increase in their activity after sodium chloride (NaCl) and magnesium chloride (MgCl_2_.6H_2_O) treatments, whereas after zinc chloride (ZnCl2) application both phages showed a significant decrease in infection.

The sequenced phage genomes are parts of a singleton cluster with sizes of 43,150 bp and 60,934 bp, respectively. Bioinformatics analyses and functional characterizations enabled the assignment of possible functions to 19 and 28 putative identified ORFs, which included phage structural proteins, lysis components and metabolic proteins.

Thirty phams were identified in both phages, 10 (33.3%) of them with known function, which can be used in cluster prediction. Comparative genomic analysis revealed significant homology between the two phages, showing the highest hits among Sf1, Sf3 and the closest *Streptomyces* phage (VWB phages) in a specific 13Kb region. However, the phylogenetic analysis using the Major Capsid Protein (MCP) sequences highlighted that the isolated phages belong to the BG *Streptomyces* phage group but are clearly separated, representing a novel sub-cluster.

**Conclusion:**

The results of this study provide the first physiological and genomic information for *S. flavovirens* phages and will be useful for pharmaceutical industries based on *S. flavovirens* and future phage evolution studies.

## Background

Bacteriophages (phages), natural viral predators of bacteria, are engaged in a constant evolutionary arms race with their hosts [1], playing major roles in the ecological balance of microbial life and in microbial diversity.

Most double-stranded DNA (dsDNA) phages share the same gene pool [2]; however, sequence comparisons reveal a widespread horizontal exchange of sequences among genomes, mediated by both non-homologous and homologous recombination. High frequency exchange among phages occupying similar ecological niches results in a high rate of mosaic diversity in local populations [3]. Studies confirm that phage genomes are mosaics and represent a large common genetic pool due to horizontal exchange [4, 5].

The screening of microbial natural products continues to constitute an important route to the discovery of chemicals for developing new therapeutic agents and evaluating the therapeutic potential of bacterial taxa [6–8]. In this respect, actinomycetes are a group of microorganisms mostly used in biotechnology for handling bioactive compounds. [9, 10]. Moreover, bacteriophages can be used to detect antiviral compound production by actinomycetes. Finally, actinophages are isolated and investigated because they can influence antibiotic production in bacterial strains, causing problems in the pharmaceutical industry. The vast majority of actinophages were isolated from sediments, but direct isolation from soil generally yields extremely low titers [11, 12]. However, although it is difficult to grow bacteriophages from soil without enrichment, a wide range of counts has been reported [13, 14].

Recently, there has been expanding interest in bacteriophages that infect *Streptomyces* species, since the phages can support the development of cloning vectors [15]. Such vectors could open the way for genetic manipulation as an important tool for *Streptomyces* improvement. Moreover, the mechanisms of the system for phage infection and multiplication could be useful in the fermentation industry and lead to the development of phage cloning vectors [16]. To date, no studies on phages isolated from *S. flavovirens*, an important source for several pharmaceutical drugs, such as actinomycin complex, mureidomycin and pravastatin [17, 18], have been carried out.

The development of high-throughput NGS (Next Generation Sequencing) technologies [19, 20] and the possibility to sequence entire genomes or transcriptomes more efficiently and economically than with first generation sequencing strategies permitted the collection of large amounts of information and the analysis of sequences from hundreds of thousands of species. Therefore, the dawn of next generation sequencing technologies has opened up exciting possibilities for whole genome sequencing in a wide range of organisms and the bacterial viruses have not been excluded from this revolution, despite the fact that their genomes are orders of magnitude smaller in size compared with bacteria and other organisms.

The Actinophage Sequence Databases (http://phagesdb.org/) currently include 5861 genomes from putative actinophages, 120 of which infect *Streptomyces* species and sixty-five of which are sequenced, but no genomes of phages isolated form *S. flavovirens* are currently available. The NCBI genome database contains around 600 Caudovirales genomes to date but the number of complete bacteriophage genomes published is growing slowly [21].

Until now, no phages belonging to *S. flavovirens* have been sequenced and relatively little is known about *S. flavovirens* phage genomics. In the present work, we report the first whole genome sequencing study and annotation of two *S. flavovirens* virulent phages. The results will provide an important genomic resource for future investigations in the bacteriophages related to *S. flavovirens* and for phage evolution studies.

## Methods

### Source of lytic actinophages

Two isolates of *Streptomyces flavovirens* phages, named Sf1 and Sf3, were obtained from the virology lab, Agric. Microbiology Department, Faculty of Agriculture, Ain Shams University, Cairo, Egypt. Phages were isolated from soil and the morphological properties were analyzed by standard methodology and reported in Marei and Elbaz (2013) [22].

### Purification of lytic actinophages

The high titer phage suspension of each isolated phage was prepared using a liquid culture enrichment technique. The high titer phage suspension of each phage was ultra-centrifuged at 30000 rpm for 90 min. at 4 °C in a Beckman L7-35 ultracentrifuge. The pellet was gently resuspended in 0.5 ml of 0.2 M phosphate buffer pH 7.2 [23].

### Adsorption rate and one-step growth experiments

The adsorption experiments were carried out with two isolated phage suspensions added to spores of their indicator host (*S. flavovirens*). Suspensions of each phage were incubated at 30 °C with gentle shaking. Samples were withdrawn at regular intervals after inoculation.

The mycelial fragments of the indicator strain were removed by centrifugation and the concentration of phage remaining in the supernatant was counted. The adsorption rates of the two phages were determined by measuring residual plaque-forming ability in membrane-filtered samples of an attachment mixture [24] and the adsorption rate constant k (mL/min) was calculated [25]. The one-step growth experiment was performed as described by Dowding (1973) [24].

### Physiochemical stability

To evaluate the phages’ stability three different chemicals (NaCl, MgCl2.6H2o and ZnCl2), were used. Five concentrations (0.1, 0.2, 0.3, 0.4 and 0.5 mM) for each salt were employed [26]. To test the effect of different treatments phage solutions for both tested strains with final concentrations of 10^7^ PFU/ml were utilized. The mixture was incubated for 10 min at room temperature (RT). The number of plaques was determined using the double layer method (plaque assay test) [27]. A control test was prepared by mixing bacterial suspension with phage without the tested chemicals.

### DNA isolation, library preparation and whole genome sequencing

Genomic DNA was isolated from the propagated phages according to the procedure described by Kieser et al. [28]. DNA quality was assessed using a Nanodrop Bioanalyzer ND1000 (ThermoScientific). Sequencing libraries were prepared by shearing 1 μg of DNA in blunt-ended fragments by linking the Ion adapters using an Ion XpressTM Plus Fragment Library Kit (Life Technologies, Carlsbad, USA) according to the manufacturer’s specifications. The sized and ligated fragments were amplified by emulsion-PCR using the Ion OneTouch 200 Template kit (Life Technologies, Carlsbad, USA). Quality and insert size distribution were assessed using an Agilent Bioanalyzer DNA 1000 chip. Libraries were sequenced on an Ion Torrent PGM semiconductor sequencer (Life Technologies, Carlsbad, USA) using the 200 bp protocol and an Ion Torrent 314 chip following the manufacturer instructions (Life Technologies, Carlsbad, USA).

### Assembly and bioinformatics analyses

Raw reads resulting from Sf1 and Sf3 sequencing were trimmed using Trimmomatic with single end mode (no quality encoding was specified to allow the program to determine it automatically [29]) and assembled separately using the gsAssembler (Roche Applied Science, Indianapolis, IN); the Graphical User Interface (GUI) version was used with the default parameters. The collected contigs were visualized and validated using Hawkeye [30]. Resulting contigs for each phage showed approximately 60-fold sequence read coverage. The expected sequence accuracy was 95% with a statistical error of less than 1 in 10,000 bp. Sequence homologies were determined by using BLASTn against the actinophage database to assign the phages to a cluster [31].

### Open reading frame (ORF) analysis and gene prediction

Open reading frames (ORFs) were identified and the genome sequences of each phage were annotated as described previously in Dobbins et al., 2004 by using DNA Master (J. G. Lawrence) (http://cobamide2.bio.pitt.edu) software and visual inspection [32]. For a genome-wide viewpoint an association with the annotation refinement, functional analysis and other explorations was developed using Phamerator. Protein sequence relationships and conserved domains within genes were also studied. Gene products were grouped into “Phamilies” generally referred to as “Phams”, or groups of proteins with a high degree of similarity to one another. The pairwise alignment scores and significant rate were determined using BLASTp and ClustalW [33].

### Genomic comparisons between the sequenced and the close related phages

Sequence comparisons were performed by using the BLAST algorithm available at NCBI [34] and Mauve software [35]. A comparison map among Sf1 and Sf3 *Streptomyces* phages and closely related phages (VWB and SV1) with available genomes in the National Center for Biotechnology Information (NCBI) nucleotide database (https://www.ncbi.nlm.nih.gov/) was generated by Circoletto (http://tools.bat.infspire.org/circoletto/) [34, 36]. For pictogram construction, bit-score values were used to describe the quality of the alignment at a given point. The bit-score is a normalized version of the score value returned by the BLAST searches, expressed in bits [37].

The phylogenetic tree of Major Capsid Protein (MCP) genes from two new isolated phages (Sf1 and Sf3) and 20 related *Streptomyces* phages available in the NCBI database was constructed with Geneious software version (R8) (http://www.geneious.com) [38] based on the Neighbor-Joining (NJ) algorithm.

## Results and discussion

### Adsorption rate constant and growth characteristics of isolated phages

Adsorption of Sf1 and Sf3 was determined using *S. flavovirens* cells grown in phage medium to the early exponential phase of growth (15-h cultures). About 82 and 85% of all infective Sf1 and Sf3 particles, respectively, were adsorbed within 20 min of contact. The adsorption reached a maximum after 30 min. for both phages. The adsorption constant K was 3.66 pL/min for Sf1 and 3.80 pL/min for Sf3, determined by the Adams’s formula [27]. The phages adsorption rates were higher than other actinophages [39], which was probably due to the strong specificity of the Sf1 and Sf3 phages to their host.

**Fig. 1:**
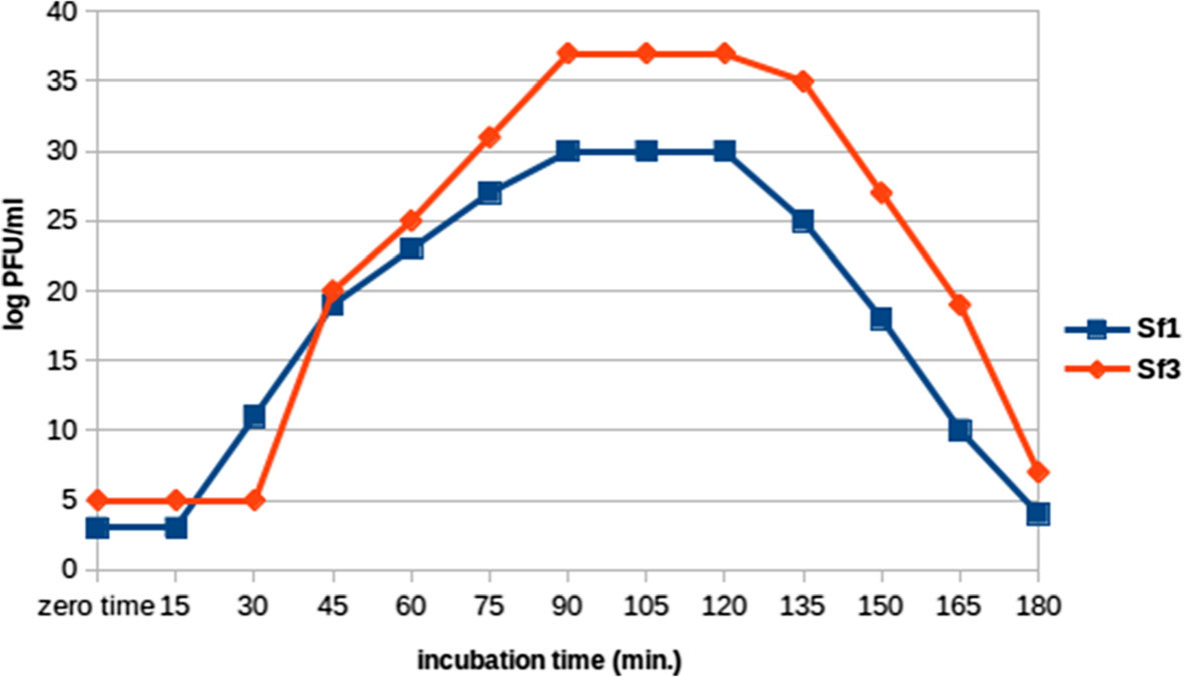
One-step growth experiment for Sf1 and Sf3 phages development on *S. flavovirens* at 30 °C

The production of Sf1 and Sf3 phages were determined in a one-step growth experiment at 30 °C. Results revealed that the latent periods of Sf1 and Sf3 were approximately 15 and 30 mins, respectively. After 30 and 45 mins the maximum rise period was shown and the burst sizes were 1.95 and 2.49 PFU/mL for Sf1 and Sf3, respectively (Fig. 1). The present results are in agreement with the data obtained from a study on 24 actinophages [40], underlining that under controlled cultural conditions the infection of isolated *Streptomycetes* cells by phages was varied.

### Physiochemical stability of isolated actinophages

**Fig. 2:**
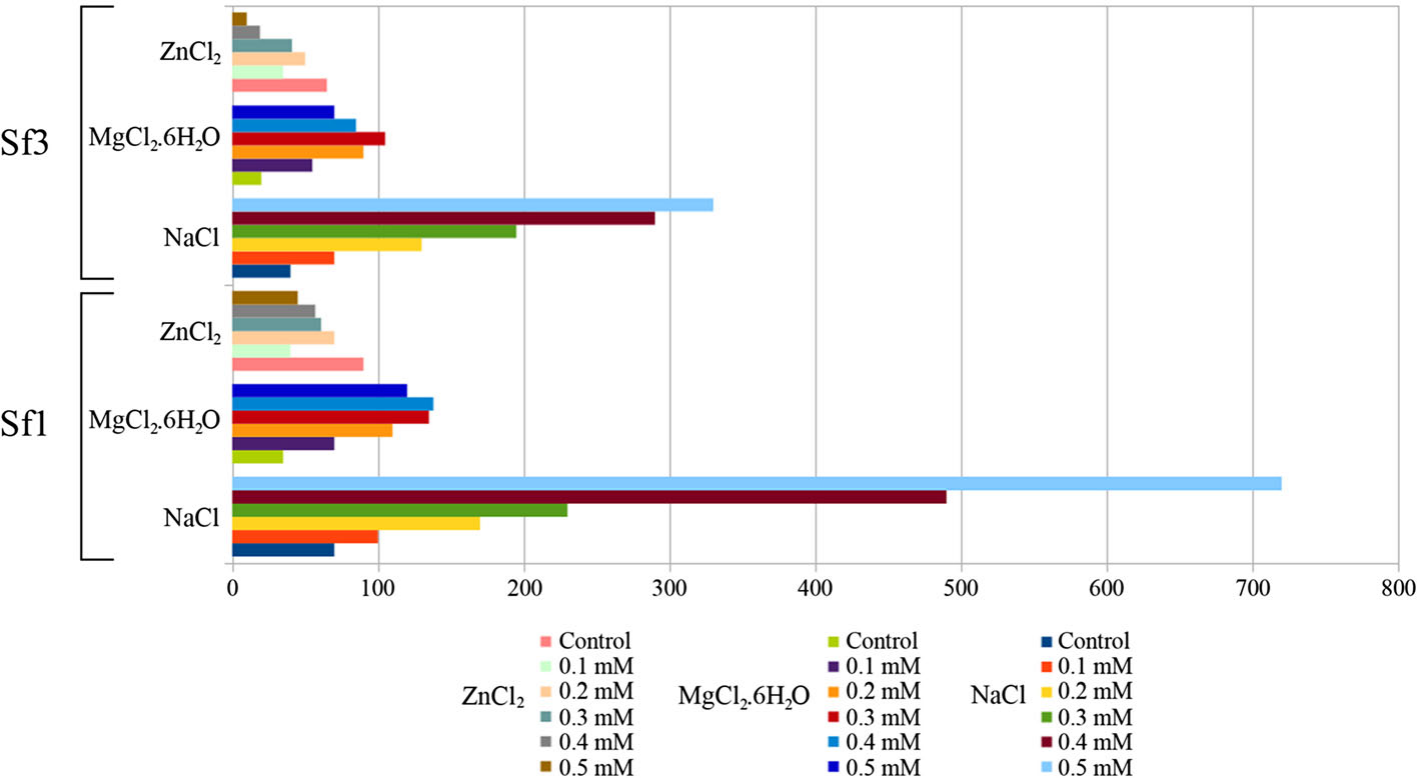
Effect of three different chemicals on the Sf1 and Sf3 infectivity

Sodium and magnesium chloride treatments yielded a significant increase in both phages’ activity for all concentrations used compared with the control, while zinc chloride application with concentrations > 0.3 mM caused a significant decrease of activity for Sf1 and Sf3 (Fig. 2). Similar results were reported in previous studies [41–43]. Absence of calcium and magnesium ions prevents adsorption and the lysis cycle, while their presence stimulates a significant increase in phage activity, probably due to the increase of adsorption and penetration rates. On the contrary, zinc and aluminum chloride showed significant loss of infectivity in both phages. This is in accordance with the experiments performed by Robert and Charles, which suggested that aluminum caused viral inactivation related to the dissociation of viral capsid proteins [44].

### Genome organization of phages Sf1 and Sf3

**Fig. 3:**
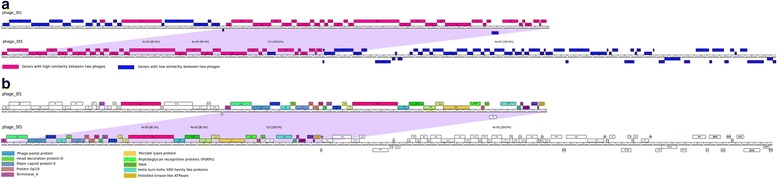
Genomic organization of Sf1 and Sf3 phages. Phages were mapped using Phamerator; the purple lines between phages underline the regions with high similarity, while the ruler corresponds to genome base pairs. The predicted genes are shown as boxes either above or below the genome (ruler), depending on whether are rightwards or leftwards transcribed, respectively. Gene numbers are shown within each box; pink boxes refereed to the genes with high similarity between two phages while the blue boxes refereed to the genes that show low similarity. **a** The phages maps showed by cluster conservation. **b** The phages maps showed by phams; genes are colored according to their function categories “phams”

Genome sequencing generated 69,719 and 107,273 reads for each phage with around 60-fold coverage and 43,150 bp, and 60,934 bp assembled sequences for Sf1 and Sf3, respectively. The pair-wise alignment [45] revealed that the genomes of Sf1 and Sf3 shared an overall high level of similarity, with conserved regions of high identity (100% identity) interspersed between regions with high variability (ranging from 23.9% to 87.5%) (Fig. 3a). A similar mosaic genome structure has been observed in most other phage genomes, indicating extensive horizontal genetic exchange among phages [46–49]. No close relatives (Singleton) from modeling of both genome construction were revealed (Fig. 1).

Annotation of Sf1 and Sf3 genomes revealed 52 and 91 putative ORFs, respectively. According to their homology, 19 out of 52 ORFs (36.5%) from Sf1 and 28 out of 91 (30.8%) from the Sf3 genome have been assigned functions compared to known conserved domains [50] (Tables 1 and 2). Isolated genes were mainly involved in DNA replication and repair, nucleotide metabolism, lysis, phage structural proteins and other enzymes. The results obtained are in agreement with other bacteriophage studies [51–53]. Phage Sf1 showed 52 ORFs (Table 1), named gp1 - gp52, while 91 ORF were identified from Phage Sf3, from gp1 to gp91 (Table 2). The majority of members of identified families are bacteriophage proteins, while others (75%) have unknown function [54, 55].

**Table 1:** Overview of Sf1 phage ORFs, summary of homology searches and annotations

ORF	Product	Strand	Begin	End	AA	Motif	Predicted functions	Homology score	E-value
ORF 1	gp1	+	126	650	175	pfam05119	Terminase_4 superfamily	65,72	1.77e-14
ORF 2	gp2	+	643	2349	569	pfam03354	Terminase_1 superfamily	243	3.32e-73
ORF 3	gp3	+	2376	3785	470	pfam05133	Phage portal protein	147	3.13e-39
ORF 4	gp4	+	3796	4842	349	cd13126	(MATE) proteins	36,9	5.07e-03
ORF 5	gp5	+	4857	5480	208	pfam09787	Golgin subfamily A5	35,58	6.35e-03
ORF 6	gp6	+	5535	6479	315	PHA00665	major capsid protein	42,17	9.33e-05
ORF 7	gp7	+	6661	7194	178	-	-	-	-
ORF 8	gp8	+	7191	7532	114	-	-	-	-
ORF 9	gp9	+	7532	7783	84	PRK14573	bifunctional D-alanyl-alanine synthetase	34,02	2.66e-03
ORF 10	gp10	+	7783	8178	132	-	-	-	-
ORF 11	gp11	+	8168	8740	191	-	-	-	-
ORF 12	gp12	+	8843	9109	89	-	-	-	-
ORF 13	gp13	+	9145	9489	115	-	-	-	-
ORF 14	gp14	+	9493	12630	1046	pfam10145	Phage-related minor tail protein	88,94	1.30e-19
ORF 15	gp15	+	12631	12837	69	-	-	-	-
ORF 16	gp16	+	12896	15148	751	-	-	-	-
ORF 17	gp17	+	15163	16281	373	pfam13550	Putative phage tail protein	43,42	1.33e-05
ORF 18	gp18	+	16281	16538	86	-	-	-	-
ORF 19	gp19	+	16563	17216	218	-	-	-	-
ORF 20	gp20	-	17411	17518	36	-	-	-	-
ORF 21	gp21	+	17716	18126	137	-	-	-	-
ORF 22	gp22	+	18141	19799	553	-	-	-	-
ORF 23	gp23	+	19824	21251	476	pfam05133	Phage portal protein	129	4.91e-33
ORF 24	gp24	+	21244	22044	267	-	-	-	-
ORF 25	gp25	+	22107	22856	250	-	-	-	-
ORF 26	gp26	+	22870	23265	132	pfam02924	Bacteriophage lambda head decoration protein D	47,27	2.61e-08
ORF 27	gp27	+	23280	24326	349	pfam03864	Phage major capsid protein E	62,35	3.13e-11
ORF 28	gp28	+	24323	24646	108	-	-	-	-
ORF 29	gp29	+	24652	25095	148	pfam09355	Phage protein Gp19	33,61	5.91e-03
ORF 30	gp30	+	25092	25445	118	-	-	-	-
ORF 31	gp31	+	25442	25726	95	-	-	-	-
ORF 32	gp32	+	25726	26127	134	-	-	-	-
ORF 33	gp33	+	26200	26865	222	-	-	-	-
ORF 34	gp34	+	26969	27292	108	-	-	-	-
ORF 35	gp35	+	27337	27765	143	-	-	-	-
ORF 36	gp36	+	27772	31344	1191	cd00254	Lytic Transglycosylase (LT)	56,65	1.82e-09
ORF 37	gp37	+	31349	32239	297	-	-	-	-
ORF 38	gp38	+	32239	33384	382	-	-	-	-
ORF 39	gp39	+	33386	34312	309	-	-	-	-
ORF 40	gp40	+	34326	34943	206	-	-	-	-
ORF 41	gp41	+	34953	36977	675	pfam12708	Pectate lyase_3 superfamily protein	73,24	8.54e-15
ORF 42	gp42	+	37059	37886	276	cd06583	Peptidoglycan recognition proteins (PGRPs)	58,45	4.03e-11
ORF 43	gp43	+	37933	38184	84	-	-	-	-
ORF 44	gp44	+	38228	38560	111	COG4467	YabA	34,76	9.25e-04
ORF 45	gp45	-	38602	39129	176	-	-	-	-
ORF 46	gp46	+	39475	40689	405	cd00093	Helix-turn-helix XRE-family like proteins	45,24	1.45e-06
ORF 47	gp47	+	40777	41046	90	-	-	-	-
ORF 48	gp48	+	41043	41237	65	-	-	-	-
ORF 49	gp49	+	41234	41752	173	-	-	-	-
ORF 50	gp50	+	41901	42383	161	-	-	-	-
ORF 51	gp51	+	42383	42481	33	-	-	-	-
ORF 52	gp52	+	42474	42923	150	cd00075	Histidine kinase-like ATPases	36,09	4.50e-04

**Table 2:** Overview of Sf3 phage, ORFs, summary of homology searches and annotations

ORF no.	Product	Strand	Begin	End	AA	Motif	Predicted functions	Homology score	E-value
ORF 1	gp1	+	16	426	137	-	-	-	-
ORF 2	gp2	+	441	2099	553	-	-	-	-
ORF 3	gp3	+	2124	3551	476	pfam05133	Phage portal protein_Gp6	129	4.91e-33
ORF 4	gp4	+	3544	4344	267	-	-	-	-
ORF 5	gp5	+	4407	5156	250	-	-	-	-
ORF 6	gp6	+	5176	5565	130	pfam02924	Bacteriophage lambda head decoration protein D	46,5	5.02e-08
ORF 7	gp7	+	5580	6626	349	pfam03864	Phage major capsid protein E	62,35	3.13e-11
ORF 8	gp8	+	6623	6946	108	-	-	-	-
ORF 9	gp9	+	6952	7395	148	pfam09355	Phage protein Gp19	33,61	5.91e-03
ORF 10	gp10	+	7392	7745	118	-	-	-	-
ORF 11	gp11	+	7748	8026	93	-	-	-	-
ORF 12	gp12	+	8026	8427	134	-	-	-	-
ORF 13	gp13	+	8500	9165	222	-	-	-	-
ORF 14	gp14	+	9269	9592	108	-	-	-	-
ORF 15	gp15	+	9637	10065	143	-	-	-	-
ORF 16	gp16	+	10072	13644	1191	pfam03864	Phage major capsid protein E	62,35	3.13e-11
ORF 17	gp17	+	13649	14539	297	-	-	-	-
ORF 18	gp18	+	14539	15684	382	-	-	-	-
ORF 19	gp19	+	15686	16612	309	-	-	-	-
ORF 20	gp20	+	16626	17243	206	-	-	-	-
ORF 21	gp21	+	17253	19277	675	pfam12708	Pectate lyase superfamily protein	73,24	8.54e-15
ORF 22	gp22	+	19359	20186	276	cd06583	Peptidoglycan recognition proteins (PGRPs)	58,45	4.03e-11
ORF 23	gp23	+	20233	20484	84	-	-	-	-
ORF 24	gp24	+	20528	20860	111	COG4467	YabA	34,76	9.25e-04
ORF 25	gp25	+	20908	21546	213	PHA03169	hypothetical protein; Provisional	35,72	5.62e-03
ORF 26	gp26	+	21757	22989	411	cd00093	Helix-turn-helix XRE-family like proteins.	45,24	1.33e-06
ORF 27	gp27	+	23077	23346	90	-	-	-	-
ORF 28	gp28	+	23343	23537	65	-	-	-	-
ORF 29	gp29	+	23534	24052	173	-	-	-	-
ORF 30	gp30	+	24201	24683	161	-	-	-	-
ORF 31	gp31	+	24668	24781	38	-	-	-	-
ORF 32	gp32	+	24774	25223	150	cd00075	Histidine kinase-like ATPases	36,09	4.50e-04
ORF 33	gp33	-	25247	25363	39	-	-	-	-
ORF 34	gp34	+	25319	25381	21	-	-	-	-
ORF 35	gp35	+	25382	26221	280	pfam00730	HhH-GPD superfamily base excision DNA repair protein	47,36	3.71e-07
ORF 36	gp36	+	26181	27680	500	-	-	-	-
ORF 37	gp37	+	27677	28327	217	cd01672	Thymidine monophosphate kinase (TMPK)	112	1.37e-30
ORF 38	gp38	+	28324	28755	144	cd04683	the Nudix hydrolase superfamily	153	3.17e-48
ORF 39	gp39	-	29387	30592	402	-	-	-	-
ORF 40	gp40	-	30712	30963	84	-	-	-	-
ORF 41	gp41	+	30962	31105	48	-	-	-	-
ORF 42	gp42	-	31162	31524	121	cd00093	Helix-turn-helix XRE-family like proteins.	41	1.99e-06
ORF 43	gp43	+	32113	32337	75	-	-	-	-
ORF 44	gp44	+	32425	32778	118	-	-	-	-
ORF 45	gp45	+	32771	33139	123	-	-	-	-
ORF 46	gp46	+	33136	33678	181	-	-	-	-
ORF 47	gp47	+	33675	33947	91	-	-	-	-
ORF 48	gp48	+	33944	34774	277	pfam12705	PD-(D/E)XK nuclease superfamily	34,99	5.68e-03
ORF 49	gp49	+	34777	35838	354	-	-	-	-
ORF 50	gp50	+	35835	36647	271	cd06127	DEDDh 3’–5’ exonuclease domain family	111	4.30e-30
ORF 51	gp51	+	36644	37099	152	-	-	-	-
ORF 52	gp52	+	37096	37713	206	cd00529	Holliday junction resolvases (HJRs)	38,38	3.22e-04
ORF 53	gp53	+	37710	37985	92	-	-	-	-
ORF 54	gp54	+	37991	38428	146	-	-	-	-
ORF 55	gp55	+	38425	38775	117	-	-	-	-
ORF 56	gp56	+	38788	39564	259	-	-	-	-
ORF 57	gp57	+	39567	40202	212	-	-	-	-
ORF 58	gp58	+	40199	40402	68	-	-	-	-
ORF 59	gp59	+	40399	40926	176	-	-	-	-
ORF 60	gp60	+	40923	41120	66	-	-	-	-
ORF 61	gp61	+	41153	41506	118	-	-	-	-
ORF 62	gp62	+	41503	42369	289	-	-	-	-
ORF 63	gp63	+	42366	42692	109	-	-	-	-
ORF 64	gp64	+	42689	42814	42	pfam10969	Protein of unknown function (DUF2771)	35,51	1.35e-04
ORF 65	gp65	+	42811	43368	186	-	-	-	-
ORF 66	gp66	+	43466	44308	281	-	-	-	-
ORF 67	gp67	-	44375	44590	72	pfam02604	Antitoxin Phd_YefM	30,73	5.22e-03
ORF 68	gp68	+	44674	46254	527	-	-	-	-
ORF 69	gp69	+	46345	47619	425	-	-	-	-
ORF 70	gp70	+	47651	47743	31	-	-	-	-
ORF 71	gp71	+	47817	47996	60	-	-	-	-
ORF 72	gp72	+	48073	48510	146	cd00397	DNA breaking-rejoining enzymes	40,54	2.27e-05
ORF 73	gp73	-	49011	49772	254	-	-	-	-
ORF 74	gp74	+	49506	49766	87	-	-	-	-
ORF 75	gp75	+	49841	49996	52	-	-	-	-
ORF 76	gp76	+	49993	50292	100	cd00085	HNH nucleases	38,22	1.45e-05
ORF 77	gp77	+	50587	51171	195	COG4983	Uncharacterized protein	79,98	9.19e-18
ORF 78	gp78	+	51278	51349	24	-	-	-	-
ORF 79	gp79	-	51714	52163	150	-	-	-	-
ORF 80	gp80	-	52167	53399	411	-	-	-	-
ORF 81	gp81	-	53709	54161	151	-	-	-	-
ORF 82	gp82	+	54647	55672	342	pfam06381	Protein of unknown function (DUF1073)	39,22	1.07e-03
ORF 83	gp83	-	55695	55889	65	-	-	-	-
ORF 84	gp84	+	55948	56844	299	TIGR01641	phage putative head morphogenesis protein	59,7	1.40e-11
ORF 85	gp85	-	56884	57198	105	PRK13502	transcriptional activator RhaR	32,72	8.64e-03
ORF 86	gp86	+	57346	57642	99	-	-	-	-
ORF 87	gp87	+	57698	57847	50	-	-	-	-
ORF 88	gp88	+	58134	58682	183	-	-	-	-
ORF 89	gp89	-	58679	59956	426	COG1783	Phage terminase_3	161	6.32e-45
ORF 90	gp90	-	60233	60691	153	-	-	-	-
ORF 91	gp91	-	60684	60932	83	-	-	-	-

### Phage structure and assembly genes

Several genes code for terminase subunit proteins, such as gp1 and 2 which code for terminase_4 (pfam05119) and terminase_1 (pfam03354) super-families, respectively. The gp3 and gp23 genes encode for the phage portal protein (pfam05133), an important protein involved in DNA transport during its packaging and ejection. Another relevant gene is gp6 which, together with gp27,codes for the major capsid protein (PHA00665) [56] and the major capsid protein E domain (pfam03864) [57], respectively, involved in the stabilization of the condensed form of DNA in phage heads. Some genes involved in tail development, gp14 (pfam10145) and gp17 (pfam13550), were also identified.

In Sf3we found a gene (gp3) encoding phage portal protein (pfam05133), crucial for DNA migration and building the junction between head and tail proteins [58], and others, such as gp7 and gb16, that encode for the major capsid protein E domain (pfam03864) [57] or for lyase (gp21), like pectate lyase_3 superfamily protein (pfam12708). A phage putative head morphogenesis protein (TIGR01641) of 110 amino acids found exclusively in phage-related proteins, was encoded by gp84. Putaive head morphogenesis proteins such as gp85, which encodesthe transcriptional activator RhaR (PRK13502), and gp89, involved in the phage terminase_3 (COG1783) synthesis, are activated during the beginning of double-stranded viral DNA packaging [59].

### DNA replication and metabolic genes

The gp44 gene encodes YabA (COG4467), a protein that interacts with the DnaA initiator and the DnaN sliding clamp and drives the control of DNA replication initiation [60, 61]. gp46 and gp52 encode for helix-turn-helix XRE-family like proteins (cd00093) [62] and histidine kinase-like ATPases (cd00075) [63], respectively, two important binding proteins with roles in the replication, repair, storage and modification of DNA. gp4 encodes a protein belonging to the MATE family (cd13126), which functions as a translocase for lipopolysaccharides [64], while gp5 codes for the golgin subfamily protein A5, a protein responsible for maintaining Golgi structure in intra-Golgi retrograde transport [65].

ORFs with the same biological roles were also identified in Sf3 phage. Indeed gp35 encodes for a HhH-GPD superfamily base excision DNA repair protein (pfam00730). This group includes endonuclease III, 8-oxoguanine DNA glycosylases and DNA-3-methyladenine glycosylase II [66]. Other members include different types of DNA and RNA exonucleases such as RNase T, oligoribonuclease, and RNA exonuclease (REX) [67]; Holliday junction resolvases (HJRs) (cd00529), endonucleases structurally similar to RNase H and Hsp70, which specifically resolve Holliday junction DNA intermediates during homologous recombination was encoded by gp52 [68]. Gp76 encodes for HNH nucleases (cd00085), an endonuclease signature which is found in viral, prokaryotic and eukaryotic proteins [69].

### Cell lysis genes

Crucial genes implicated in lysis activities, such as the cell wall degradation process in bacteria during host infection, were identified in the Sf1 genome. Indeed, gp36 encodes for the lytic transglycosylase (LT) (cd00254) that catalyzes the cleavage of the beta-1,4-glycosidic bond between N-acetylmuramic acid and N-acetyl-D-glucoseamine, similar to “goose-type” lysozymes. gp42 encodespeptidoglycan recognition proteins (PGRPs) (cd06583), namely receptors that bind and hydrolyze peptidoglycans of bacterial cell walls, and contains two conserved histidines and a cysteine, typical residues of zinc binding sites [70].

While gp21 is included in the pectate lyase superfamily (pfam12708), proteins with a beta helical structure like pectate lyase and most closely related to glycosyl hydrolase family and gp22 encodes to Peptidoglycan recognition proteins (PGRPs) (cd06583) [70], were identified in Sf3 genome.

Both phage genomes show up to bring a modular organization, with genes of related function clustered together (Fig. 3a and b). DNA sequences of the first 13 kb in Sf3 are highly similar to the last DNA sequences in Sf1 and encode for DNA packaging structural proteins (Fig. 3b).

On the basis of the amino acid sequence similarity between the gene products, the conserved pfam05133 motif and the gene locations, orf3 is predicted to encode a portal protein in both phages. No small terminase-encoding gene could be identified in either genome. The largest gene in Sf1 genome is located in orf36 (3.5 kb) encoding the lytic transglycosylase (LT), while the largest one in Sf3 genome with the same length is orf16, encoding the major capsid protein E domain. [48, 71, 72]. A possible lyase gene is positioned distinctively in both phage genomes (orf41 for Sf1 and orf21 for Sf3). Those genes located downstream in both phage genomes encode proteins involved in DNA synthesis, metabolism and repair (Fig. 3b).

### Evolutionary relationship of Sf1 and Sf3

Sf1 and Sf3 phages show 30 phams, where 29 out of 30 phams contain two members (Table 3), while three members belong to pham number 12. Ten phams (33.3%) were assigned with known functionality; the others are unknown. Therefore, some of these phams are informative and can be used in evolutionary studies. Indeed, as reported for mycobacteriophages [73], single, ubiquitous, semi-conserved genes can be utilized for cluster prediction, useful when the whole genome sequence is unavailable. The 30 identified phams, which include important genes (see below), underline a close phylogenetic relationship between the two isolated phages and provide important information that can be used in future evolutionary relationship studies by comparing the genes identified in the *Streptomyces flavovir*ens phages and homologous genes in other bacteriophages.

**Table 3:** Related Conserved Domains (CD) to the detected Phamilies using Phamerator

Pham	Conserves Domains (CD)	Number of members	Mean translation length	Phage Sf1	Phage Sf3
1	-	2	136	ORF 21	ORF 1
2	-	2	552	ORF 22	ORF 2
3	Phage portal protein	2	475	ORF 23	ORF 3
4	-	2	266	ORF 24	ORF 4
5	-	2	249	ORF 25	ORF 5
6	Bacteriophage lambda head decoration protein D	2	130	ORF 26	ORF 6
7	Phage major capsid protein E	2	348	ORF 27	ORF 7
8	-	2	107	ORF 28	ORF 8
9	Phage protein Gp19	2	147	ORF 29	ORF 9
10	-	2	117	ORF 30	ORF 10
11	-	2	93	ORF 31	ORF 11
12	Terminase_4 superfamily	3	132,3333	ORF 1, ORF 32	ORF 12
13	-	2	221	ORF 33	ORF 13
14	-	2	107	ORF 34	ORF 14
15	-	2	142	ORF 35	ORF 15
16	-	2	296	ORF 37	ORF 17
17	-	2	381	ORF 38	ORF 18
18	-	2	308	ORF 39	ORF 19
19	-	2	205	ORF 40	ORF 20
20	Pectate lyase superfamily protein	2	674	ORF 41	ORF 21
21	Peptidoglycan recognition proteins (PGRPs)	2	275	ORF 42	ORF 22
22	-	2	83	ORF 43	ORF 23
23	YabA	2	110	ORF 44	ORF 24
24	Helix-turn-helix XRE-family like proteins	2	407	ORF 46	ORF 26
25	-	2	89	ORF 47	ORF 27
26	-	2	64	ORF 48	ORF 28
27	-	2	172	ORF 49	ORF 29
28	-	2	160	ORF 50	ORF 30
29	-	2	34,5	ORF 51	ORF 31
30	Histidine kinase-like ATPases	2	149	ORF 52	ORF 32

orf27 (Sf1) and orf7 (Sf3) as members of pham n.7 were assigned as phage major capsid protein (MCP) E domains; this important class of genes was also used as a single gene prediction system for the mycobacteriophage clusters analysis [73]. orf23 (Sf1) and orf3 (Sf3), members of pham n. 3, were classified as phage portal proteins. These proteins were used in some previous investigations as a marker of diversity indicating, in some cases, the connections between habitat properties, microbial community structure and phage community composition [74]. orf29 (Sf1) and orf9 (Sf3) are the members of pham n.9, were assigned to phage protein gp19, an important tail component. Most of the proteins forming the phage tail components as well as other needle-like assemblies (e.g. secretion systems and bacteriocins) have a common origin from a single protein module [74]. This evidence emphasizes the importance of phage protein diversification and specialization in the evolution of different and complex bacterial systems and in bacterial adaptation, developing new functions and providing a distinct selective advantage [74].

As expected, the virulent phages developed phams involved in lysogenic pathways. Indeed, orf41 (Sf1) and orf21 (Sf3), grouped in pham n.20, showed high homology to the pectate lyase superfamily protein that can modify the properties of polysaccharides. Since the pectinolytic protein family is commonly represented in prokaryotic and eukaryotic microorganisms and, in plants, is involved in remodelling cell walls, it is clear that the divergence from the ancestral protein over time has allowed different micro-organisms to target a range of pectin-like substrates while the overall structure has been maintained [75]. orf42 (Sf1) and orf22 (Sf3) are members of pham n.21 and classified as peptidoglycan recognition proteins (PGRPs), an innate class of immunity molecules present in insects, mollusks, echinoderms, and vertebrates that by interacting with peptidoglycan in the cell wall, rather than permeabilizing bacterial membranes, kills bacteria. These proteins were reported, at least in one carboxy-terminal domain, as homologous in bacteriophage and bacteria [76]. orf46 (Sf1) and orf26 (Sf3) are grouped in pham n.24 and were identified as helix-turn-helix (HTH) XRE-family-like proteins, one of the early studied regulatory DNA-binding proteins involved in metabolic regulation in bacteria. This class of genes encodes components to process environmental metabolites (e.g. lactose) and to produce interacting constituents in the development of a lytic or lysogenic pathway in phages. A common ancestor for all DNA-binding domains was suggested and, through its duplication and divergence, the diversity of transcription regulators that drive bacterial and phage genes was generated. The HTH fold investigations confirmed the significance of this module in DNA–protein interactions across a wide phylogenetic spectrum including a wide variety of phages [77].

orf26 (Sf1) and orf6 (Sf3), members of pham n. 6, were classified as bacteriophage lambda head decoration protein D. Since the protein allows for the display of many copies of a foreign protein, which is advantageous for displaying weak ligands for affinity selection, a useful platform for phage polypeptide display was recently developed [78]. Interestingly, orf32 in Sf1 and orf12 in Sf3 were not assigned functions previously, although they belong to the pham n. 12 together with orf 1 (Sf1) which is classified as terminase_4.

**Fig. 4:**
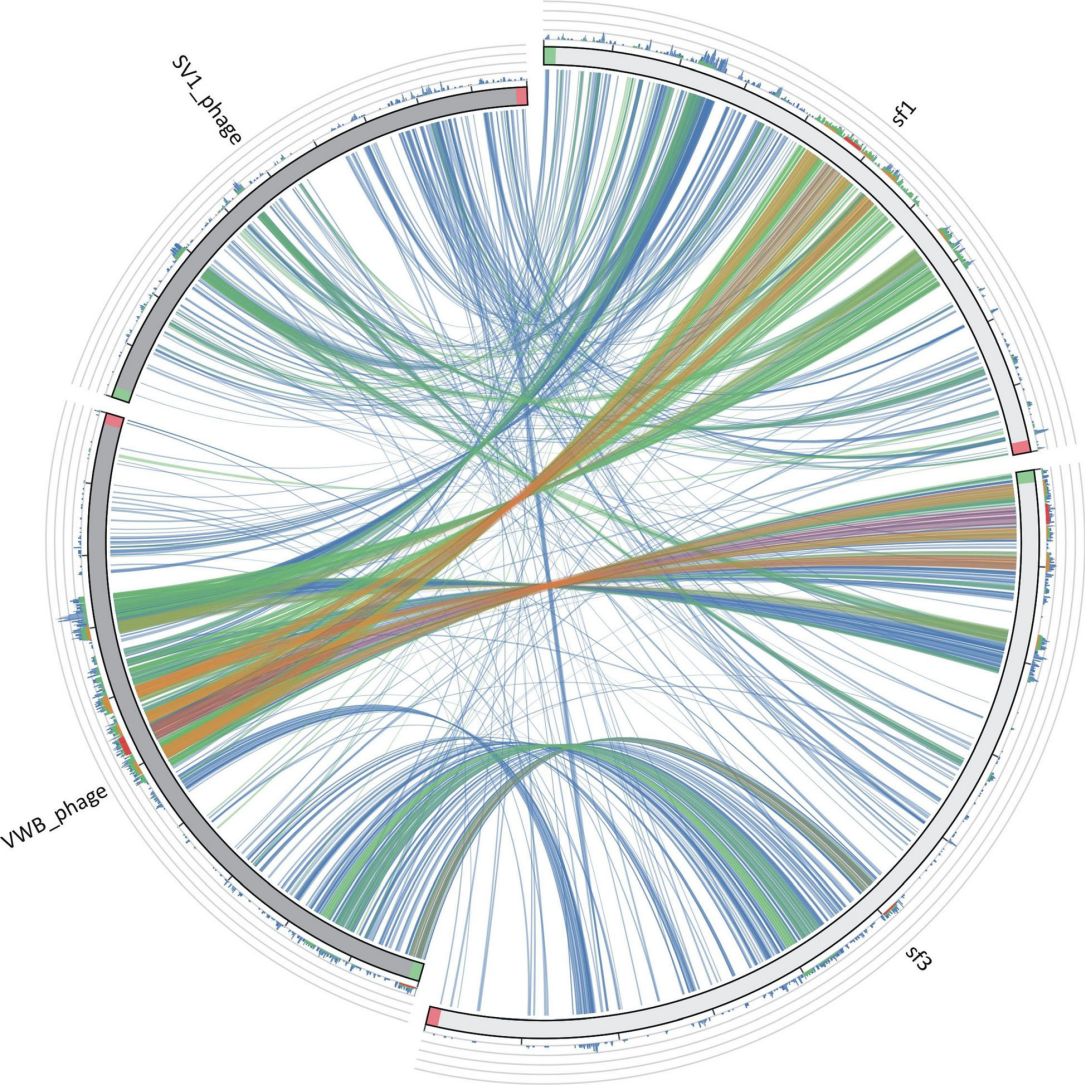
Sequence similarities among Sf1, Sf3, VWB and SV1 phages. The picture shows the results of the BLAST local alignments using Sf1 and Sf3 as a query against the VWB and SV1 phages sequences. The different colours (blue, green, orange and red) represent the overall quality of the aligned segments along the phage sequences, evaluated on the basis of the bit-score values from the worst to the best score (blue to red). The bit-score is a normalized version of the score value obtained by BLAST searches, expressed in bits. The height of the coloured bars in the histogram shows how many times each colour hits a specific fragment of the other phage sequences. A twist in a ribbon indicates that the local alignment is inverted (query and database sequence on opposite strands)

A standard Nucleotide NCBI BLAST (blastn) search was developed using both Sf1 and Sf3 phage whole genome sequences as a query against a non-redundant nucleotide sequences database. Starting from a whole phage dataset (https://www.ncbi.nlm.nih.gov/) the available phage genomes with the best identity percentages (VWB and SV1) were chosen and a pictogram was developed (Fig. 4). Seventy-eight percent identity for both *S. flavovirens* phages compared to the complete genome of bacteriophage VWB, isolated from *S. venezuelae* strain ETH 14630 (AY320035.2), was exhibited (with 29% and 36% of coverage for Sf1 and Sf3, respectively), while 75% of identity for both studied phages with *S. venezuelae* phage SV1 (JX182371.1) was reported, but with low query coverage (11% for Sf1 and 14% for Sf3), probably due to the phylogenetic distance between the compared phages.

The alignment of both Sf1 and Sf3 genomes against the sequences of VWB phage, carried out by Mauve software, revealed that most hits occurred around a 13Kb region (Fig. 4). The approximate location of this region were (18000–31000) within the Sf1 genome, (1–13000) in the Sf3 genome and (23000–36000) in the VWB genome. On the contrary, the alignment of both *S. flavovi*rens phage genomes versus the sequences of SV1 showed only a short region (~1Kb) with moderate bit score ranging from 9691–10707 and 10300–11208 in the genomes of Sf1 and Sf3, respectively, consistent with the low sequence coverage obtained.

**Fig. 5:**
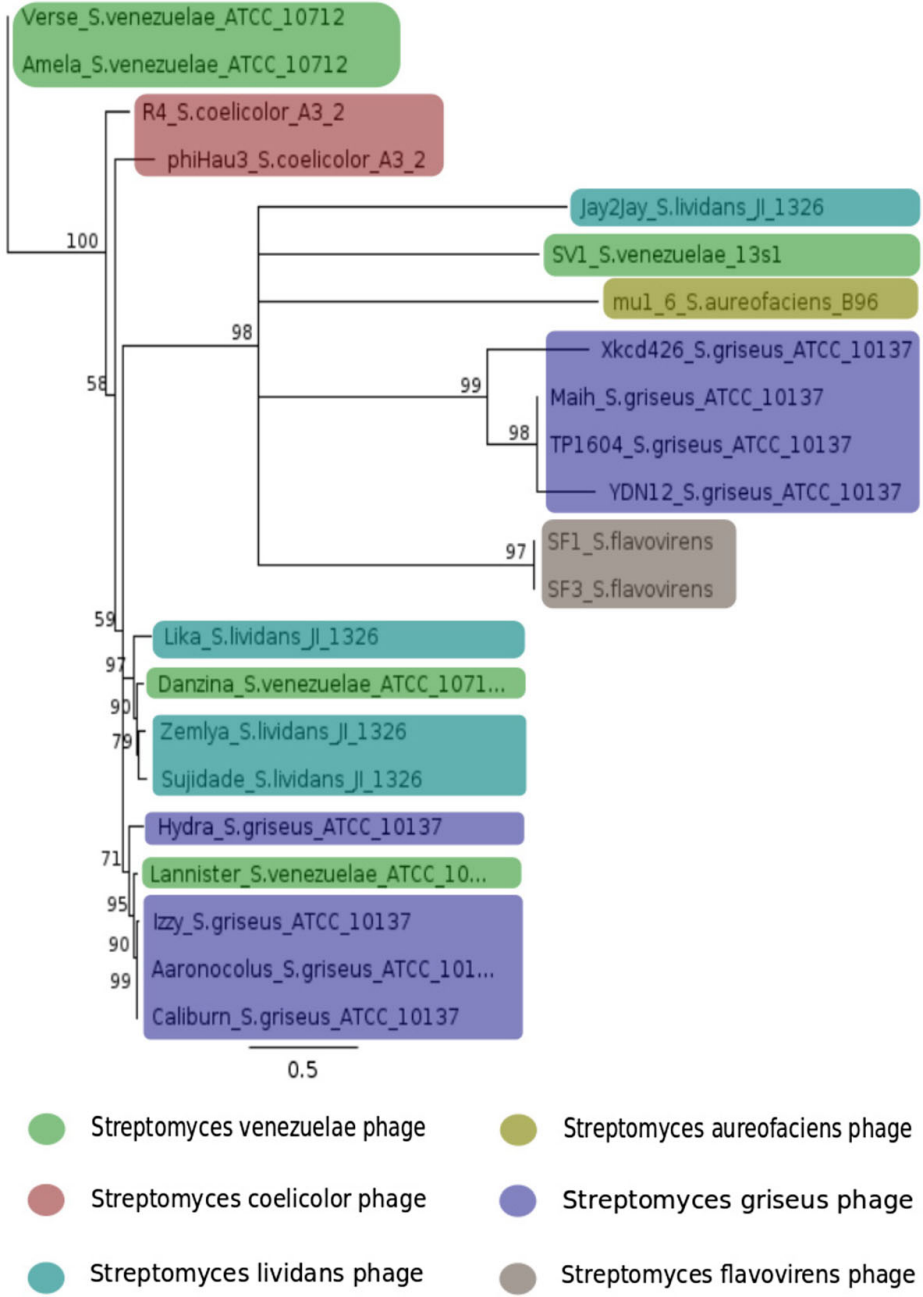
Phylogenetic analysis of studied phages and other members (20) of the Streptomyces phages group based on MCPs. Bootstrap values indicate the number of times a node was supported in 1000 resampling replications

The MCPs diversity between Sf1, Sf3 and 20 related *Streptomyces* phages, due to a combination of illegitimate and homologous recombination [79] and mutational drift, was also evaluated. The current investigation highlighted the hybrid generation between phage genera [80] or phage families [81]. Twenty-two *Streptomyces* phages were grouped in five main branches (Fig. 5). The Lannister MCP shared a close evolutionary relationship with the Izzy, Aaronocolus, and Caliburn sequences, demonstrating that phages may undergo genetic exchange by horizontal gene transfer from a large shared pool [4] and that horizontal gene transfer between phages is a component of their evolution. Numerous gene exchanges within each major clade and core phage functions do not appear to have co-evolved with specific hosts [82].

Our phylogenetic analysis is useful for further studies, since both Sf1 and Sf3 were recovered in a clade that included phages that infect *Streptomyces* species but most of these phages (Maih, YDN12, Xkcd426 and TP1604) were members of the BG phage cluster; this clustering does not represent a phylogenetic or taxonomic grouping but rather provides a framework for reflecting their overall genome relationships and for identifying genes that have been recently exchanged and their genomic context [83, 84]. Moreover, Sf1 and Sf3 grouped in a separate branch, indicating that isolated phages belong to the BG phage cluster but represent a different sub-cluster.

## Conclusion

Recently, large advances have occurred in phage genomics; nevertheless,the full extent of phage diversity and evolutionary pathways are yet unknown. With the advent of NGS technologies a much greater volume of transcriptome and genome sequences is available and we can therefore expect an increased flow of new data in upcoming years. Current assessment suggests that more than 1031 phages exist on earth, representing more than ten million phage “species”. Of these, less than 6000 have been observed using electron microscopy and fewer than 1000 genomes have been sequenced. The available sequences show that the majority of phages analyzed are tailed phages belonging to the family Siphoviridae, but less is known about the degree of their genetic diversity. The genomic characterization of phages is necessary to evaluate their important ecological impact. In spite of their ubiquity, phages have not yet been characterized for many bacterial genera. In the present study, biological, physiochemical and genome sequences of two new virulent *Streptomyces* phages are presented, representing the first genomic report of *S. flavovirens* phages which may represent a new sub-cluster of the BG *Streptomyces* phage cluster.

## Abbreviations

dsDNA: Double-stranded DNA
HJRs: Holliday junction resolvases
HTH: Helix-turn-helix
LT: Lytic transglycosylase
MCP: Major capsid protein
NCBI: National Center for Biotechnology Information
NGS: New Generation Sequencing
ORFs: Open reading frames
PGRPs: Peptidoglycan recognition proteins
Phages: Bacteriophages
REX: RNA exonuclease
TDP: Thymidine diphosphate
TMP: Thymidine monophosphate
TMPK: Thymidine monophosphate kinase
TTP: Thymidine triphosphate
